# Novel Operation on the Eye

**Published:** 1896-12

**Authors:** 


ARTICLE III.

### NOVEL OPERATION ON THE EYE.

Professor Deutschmann is one of the best known and most eminent oculists of Germany, and among the promiment oculists of the United States are many young men who were his pupils.

For over a year he has been experimenting in transfusions of liquid from the eye of a living rabbit to that of a human being. All of this work was, however, performed by the Hamburg oculist in the privacy of his laboratory.

It is only now, when the success of his daring and novel experiment has been demonstrated, that he consents to disclose the simple facts. The first news of his discovery reached the oculists of the United States through a brief special cablegram from Berlin on July 30.

It stated that Professor Deutschmann had cured cases of blindness caused to the retina by substituting parts from the eye of a rabbit. This statement was received with incredulty by the oculists of New York.

Up to the present time there are six people in Hamburg whose eyesight have been saved by Professor Deutschmann through infusion from the eye of a living rabbit. Upon one of these patients the operation was tried successfully on both eyes.

This makes seven operations in all performed by Professor Deutschmann according to the new method, and in each case it has been a success. The patients, who had been nearly blind, and who were certainly threatened with final loss of the diseased organ, have now been enjoying the best of eyesight since the operation was performed.

From four to twelve months have elapsed in each of these cases. There has been a steady improvement since the time of the operation.

Professor Deutschmann thought it best to wait this length of time before disclosing any of the facts, so as to be assured that there would be no final relapse. The cure, instead of being temporary, is now regarded as permanent, and this new and surprising operation will probably soon spread to all parts of the earth.

As described by the assistant of Professor Deutshmann, the new operation is as simple as it is startling. Professor Deutschmann does not attempt to replace a diseased retina nor does he claim that he can substitute the retina from a rabbit for that of a human being.

At the same time he does not hesitate to operate on the retina itself by making such incisions through it as may be necessary to assure the success of his process. He has found that blindness frequently ascribed to disease of the retina is in reality often caused by a detachment of the latter.

Such detachment and shrinkage of this delicate membrane result from absorption of the vitreous humor which constitutes nearly nine-tenths of the body of the eye itself. This vitreous humor is enclosed in a sac, making what is commonly called the eye ball, and through it the image passes from the lens to the retina.

The latter is retained in place by the full rounded body of the sac of vitreous humor loses its normal volume through absorption, the retina stretched around it skrinks, having no support of its own, and thus the sight is finally destroyed.

A great number of disorders of the eye belong to this class. Blood diseases, hemorrhages, excessive mental work and nervous disorders are some of the initial causes leading to absorption of the vitreous humor, with consequent detachment or skrinkage of the retina and resulting blindness. The great discovery which Professor Deutschmann has made is that the deficiency of vitreous humor in the human eye can now be supplied from the eye of a living rabbit.

The transfusion of vitreous humor from the eye of the rabbit to the eye of the patient on the oculist's operating table is accomplished through a simple rubber tube. Before this is done, however, the most delicate kind of eye surgery is necessary.

The operating surgeon cuts into the sac of vitreous humor in the eye of his patient with a steel canula. This cuts through the sclerotic, through the choroid and through the retina.

The eye-ball of the patient is turned down so as to expose as much of the top surface as possible. The cutting is done from the top, in a place that is never seen under normal conditions.

An assistant holds a live rabbit, and with the steel canula he cuts deep into the sac of vitreous humor in its eye, which almost exactly corresponds with the eye of a human being in its construction and texture.

The eyes of rabbit and patient are then brought as close together as possible. A small rubber tube is introduced into both incisions, being pushed deep into the sac of vitreous humor in either case.

A gentle pressure of the finger upon the eye of the rabbit is then sufficient to pump the vitreous humor into the rubber tube and thence into the humor sac of the patient. In less than a minute the deficient quantity of vitreous humor can be made up from the eye of the living rabbit.

As the sac of vitreous humor in the human eye swells out with the infusion of a new liquid, the retina expands correspondingly. Thus it reaches its normal state, and the complete eyesight is at once restored.

To keep the eye in this condition is now the object of the surgeon. The rubber tubing is at once removed and the healing of the wound begins.

It will be seen that this astonishing operation does not in any way effect the appearance of the eye, and that no solid substance is transferred from the eye of the rabbit to that of the patient. What the rabbit loses is simply a transparent liquid, constituting about nine tenths of its eye.

The whole operation can be performed in a very few minutes. In transfusions of blood a tube with a pumping bulb is used by surgeons. But this new operation on the eye is so delicate and the quantity of liquid passed through the tube so small that it is believed no bulb is necessary. Holding the little rubber tube between his fingers, the oculist can easily press the liquid into the eye of the patient.

Detachment of the retina follows the loss of a small quantity of the vitreous humor in the human eye, and blindness results when less than half of it has been absorbed. It is not claimed by Professor Deutschmann that cases of total blindness resulting from loss of the vitreous humor can be cured by his new method. Thus far the operation has only been resorted to for the purpose of averting such blindness.—*Exchange.*

